# Plectin 1d Isoform as a Potential Regulator of Metastatic Progression in Papillary Thyroid Carcinoma

**DOI:** 10.3390/ijms27146339

**Published:** 2026-07-16

**Authors:** Hulya Gundesli, Betul Budak, Kazim Yalcin Arga

**Affiliations:** 1 Department of Medical Biology, Gulhane Faculty of Medicine, University of Health Sciences, Ankara 06010, Türkiye; 2 Department of Bioengineering, Faculty of Engineering, Marmara University, Istanbul 34854, Türkiye; betul.budak@marmara.edu.tr (B.B.); kazim.arga@marmara.edu.tr (K.Y.A.); 3 Center for Nanotechnology & Biomaterials Applications and Research, Marmara University, Istanbul 34854, Türkiye; 4 Health Biotechnology Joint Research and Application Center of Excellence, Istanbul 34220, Türkiye

**Keywords:** papillary thyroid carcinoma, *PLEC 1d*, metastasis, HEY1

## Abstract

Papillary thyroid carcinoma (PTC) typically exhibits a favorable prognosis; however, lymph node and distant metastases continue to present significant clinical challenges. Plectin isoform 1d (*PLEC 1d*) contributes to myofiber integrity and is implicated in cytoskeletal organization and cancer cell motility, but its role in PTC progression is largely unknown. In this study, *PLEC 1d* expression was examined by RT-PCR in three PTC cell lines (MDA-T32, MDA-T41, MDA-T120) and normal thyroid cells (Nthy-ori-3-1). CRISPR-Cas9-mediated knockdown (KD) of *PLEC 1d* was performed in MDA-T41 cells, with KD efficiency confirmed by RT-qPCR. Cell proliferation and migration were assessed using EdU incorporation and wound-healing assays, respectively. RNA sequencing (RNA-seq) combined with RT-qPCR and Western blotting was employed to identify downstream targets. Accordingly, *PLEC 1d* mRNA and total plectin protein levels were substantially elevated only in MDA-T41 cells. *PLEC 1d* KD reduced proliferation by 6.6% and migration by 26%. Transcriptomic analysis revealed 170 differentially expressed genes, including significant downregulation of *LAMA4*, *MMP1*, and *HEY1*. HEY1 downregulation was confirmed at both mRNA and protein levels. Although *LAMA4* and *MMP1* were also downregulated following *PLEC 1d* KD, *LAMA4* mRNA expression varied across PTC cell lines, suggesting cell-line-specific regulation. Notably, *MMP1* was consistently upregulated at the mRNA level in all PTC cell lines, while HEY1 showed elevated expression at both the transcript and protein levels. Collectively, these findings indicate that *PLEC 1d* enhances cell migration and may contribute to cellular processes associated with metastatic behavior in a subset of PTC cells, potentially through a HEY1-dependent mechanism.

## 1. Introduction

Thyroid cancer (THCA) is among the most common malignant endocrine tumors, occurring in women about four times more often than in men [1]. It represents nearly 2.5% of all malignancies and accounts for approximately 95% of endocrine tumors worldwide [2].

Thyroid cancers are classified into four main subtypes: papillary, follicular, anaplastic, and medullary carcinoma [3]. Papillary thyroid carcinoma (PTC) is the most prevalent, comprising over 80% of cases globally [3]. PTC generally has a favorable prognosis, with a 10-year survival rate above 95% [4].

Although PTC generally has a favorable prognosis, its onset is often clinically silent. Most patients do not exhibit specific symptoms, and the disease is frequently detected through nodular changes in the cervical thyroid region [5]. Differentiating benign from malignant thyroid nodules remains a significant clinical challenge [5]. As a result, lymph node metastasis may already be present at diagnosis in some patients [6]. Studies have shown that patients with lymph node metastasis have a higher risk of distant metastasis than those without nodal involvement [7]. While most PTC patients experience favorable outcomes, tumor recurrence and metastasis continue to impact treatment efficacy and patient survival [8]. Thus, identifying effective biomarkers for early diagnosis and improved prognostic evaluation is essential.

Our previous pan-cancer study utilized transcriptomic data from The Cancer Genome Atlas (TCGA) database to examine the expression patterns and potential diagnostic and prognostic significance of the *PLEC* gene (NM_000445) and its eight transcript isoforms (*PLEC 1*; NM_201380.4, *PLEC 1a*; NM_201384.3, *PLEC 1b*; NM_201382.4, *PLEC 1c*; NM_000445.5, *PLEC 1d*; NM_201381.3, *PLEC 1e*; NM_201379.3, *PLEC 1f*; NM_201378.4, and *PLEC 1g*; NM_201383.3) across 12 cancer types [9]. The results demonstrated that the *PLEC 1d* isoform showed significantly elevated expression across all four stages of THCA and exhibited a higher AUC value, highlighting its potential as a promising diagnostic biomarker for thyroid cancer [9]. Additionally, analysis of the TCGA dataset indicated that most thyroid cancer samples corresponded to PTC.

Studies have shown that plectin isoforms have tissue-specific functions. Thus, Plectin 1d is highly expressed in muscle tissue and serves as a key sarcomeric component associated with Z-disks [10,11]. In addition, Plectin isoforms 1, 1d, 1e, 1f, and 1g localize to podosomes and support cell motility in SW480 colon carcinoma cells [12]. In cancer cells, podosomes are called invadopodia, which are actin-based protrusions that degrade the extracellular matrix and promote invasion and migration [13,14].

Alterations in total *PLEC* expression, without consideration of specific isoforms, have been associated with the development of various cancer types [15,16,17,18,19,20,21,22]. Increasing evidence indicates that plectin contributes to cancer cell proliferation, invasion, and migration [12,19,21,22,23,24,25]. However, most research has focused on the *PLEC* gene as a whole rather than its individual isoforms. Notably, Bausch et al. identified the *PLEC 1* isoform as a potential biomarker for primary and metastatic pancreatic ductal adenocarcinoma (PDAC) [26]. Additionally, *PLEC 1a* is highly expressed in triple-negative breast cancer cells and promotes invasive migration [27]. The potential roles of *PLEC 1d* and other isoforms in cancer development, however, remain largely unexplored.

To date, there is a lack of effective diagnostic markers for PTC patients with lymph node metastasis. Therefore, the present study focuses on elucidating the function of the *PLEC 1d* isoform in PTC progression and cell migration.

## 2. Results

### 2.1. Differential Expression of PLEC 1d Isoform Across PTC Cell Lines

**Figure 1:**
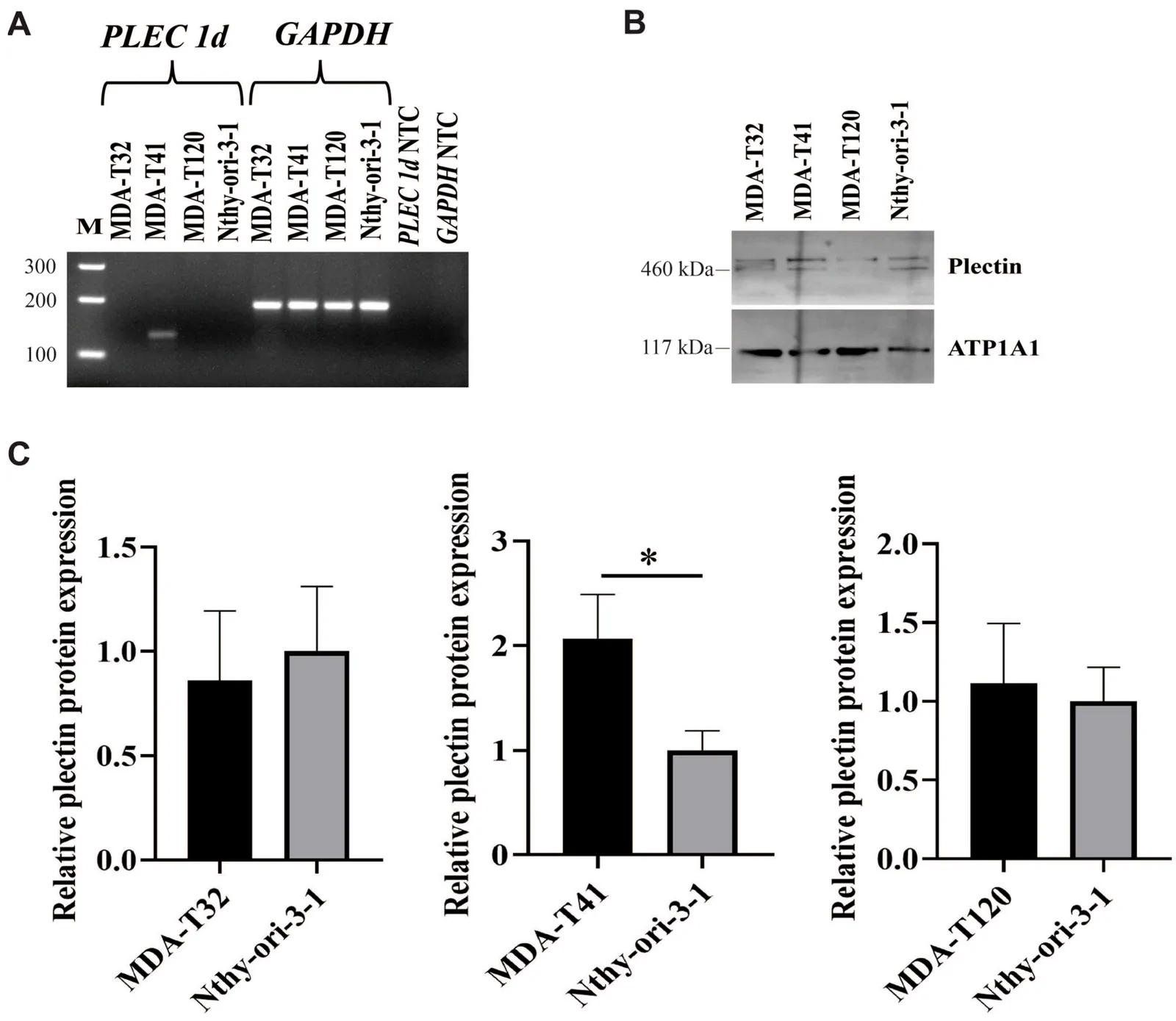
*PLEC 1d* mRNA and total plectin protein expression in PTC cell lines. (**A**) RT-PCR demonstrating *PLEC 1d* (129 bp) and *GAPDH* (185 bp) mRNA expression in PTC cell lines. NTC: non-template control. M: 100 bp molecular weight marker. (**B**) Western blot showing total plectin protein (~500 kDa) expression in PTC cell lines, with ATP1A1 (100–110 kDa) as the loading control. (**C**) Quantification of relative plectin protein expression in PTC cell lines normalized to the normal thyroid cell line Nthy-ori-3-1. * *p* < 0.05.

In this study, RT-PCR analysis demonstrated that *PLEC 1d* mRNA expression was not detected in MDA-T32 and MDA-T120 PTC cell lines, as well as in the normal thyroid cell line Nthy-ori-3-1 (Figure 1A, Supplementary Figure S1). In contrast, significant *PLEC 1d* mRNA expression was observed in the MDA-T41 PTC cell line (Figure 1A, Supplementary Figure S1A).

Western blot analysis revealed a significant increase in total plectin protein expression in the MDA-T41 PTC cell line (Figure 1B,C, Supplementary Figure S1B), whereas no significant difference in total plectin expression was detected in MDA-T32 and MDA-T120 compared to Nthy-ori-3-1 (Figure 1B,C, Supplementary Figure S1B).

### 2.2. Functional Impact of PLEC 1d Knockdown on Proliferation and Migration in MDA-T41 PTC Cells

**Figure 2:**
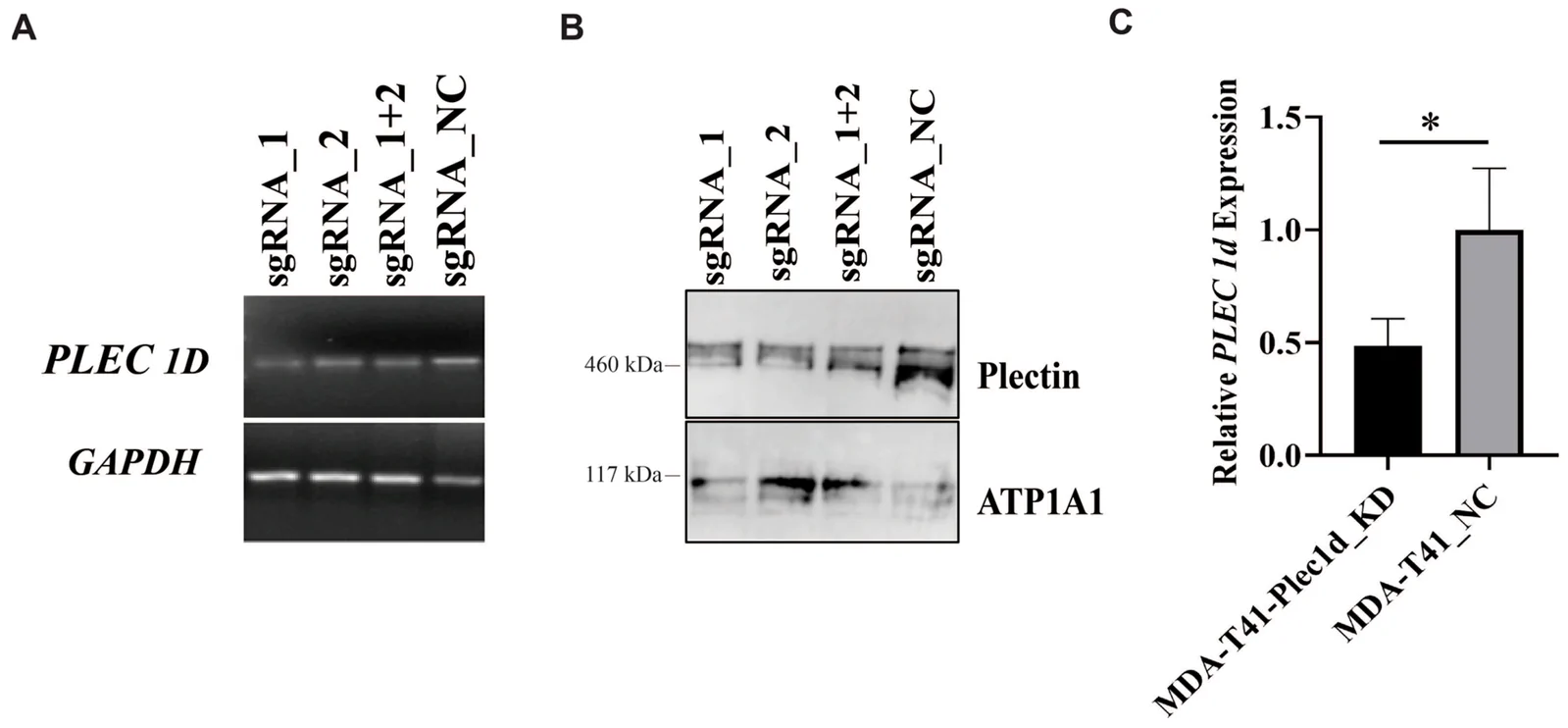
*PLEC 1d* KD validation in MDA-T41 PTC cells. (**A**) RT-PCR showing *PLEC 1d* mRNA expression following transfection with sgRNA_1, sgRNA_2, or their combination (sgRNA_1+2). *GAPDH*: loading control; NC: non-targeting control sgRNA. (**B**) Western blot confirming reduced total plectin protein (~500 kDa) expression under the same conditions, with ATP1A1 (100–110 kDa) as the loading control. (**C**) RT-qPCR quantification of *PLEC 1d* mRNA levels in sgRNA_1+2-treated cells relative to NC, demonstrating a mean KD efficiency of 52.5% ± 1.78% (SD, *n* = 3). * *p* < 0.05.

In this study, the *PLEC 1d* transcript isoform was suppressed in the MDA-T41 cell line using a CRISPR-Cas9-mediated approach. Two distinct sgRNAs were included in the transfection cocktail, resulting in at least a 50% reduction (52.5% ± 1.78%) in *PLEC 1d* mRNA expression (Figure 2A–C). However, no statistically significant expression alterations were detected for the remaining plectin isoforms (*PLEC 1*, *1a*, *1b*, *1c*, *1e*, *1f* and *1g*) when comparing MDA-T41-plec1d_KD and MDA-T41-plec1d_NC cells (Supplementary Figure S2).

**Figure 3:**
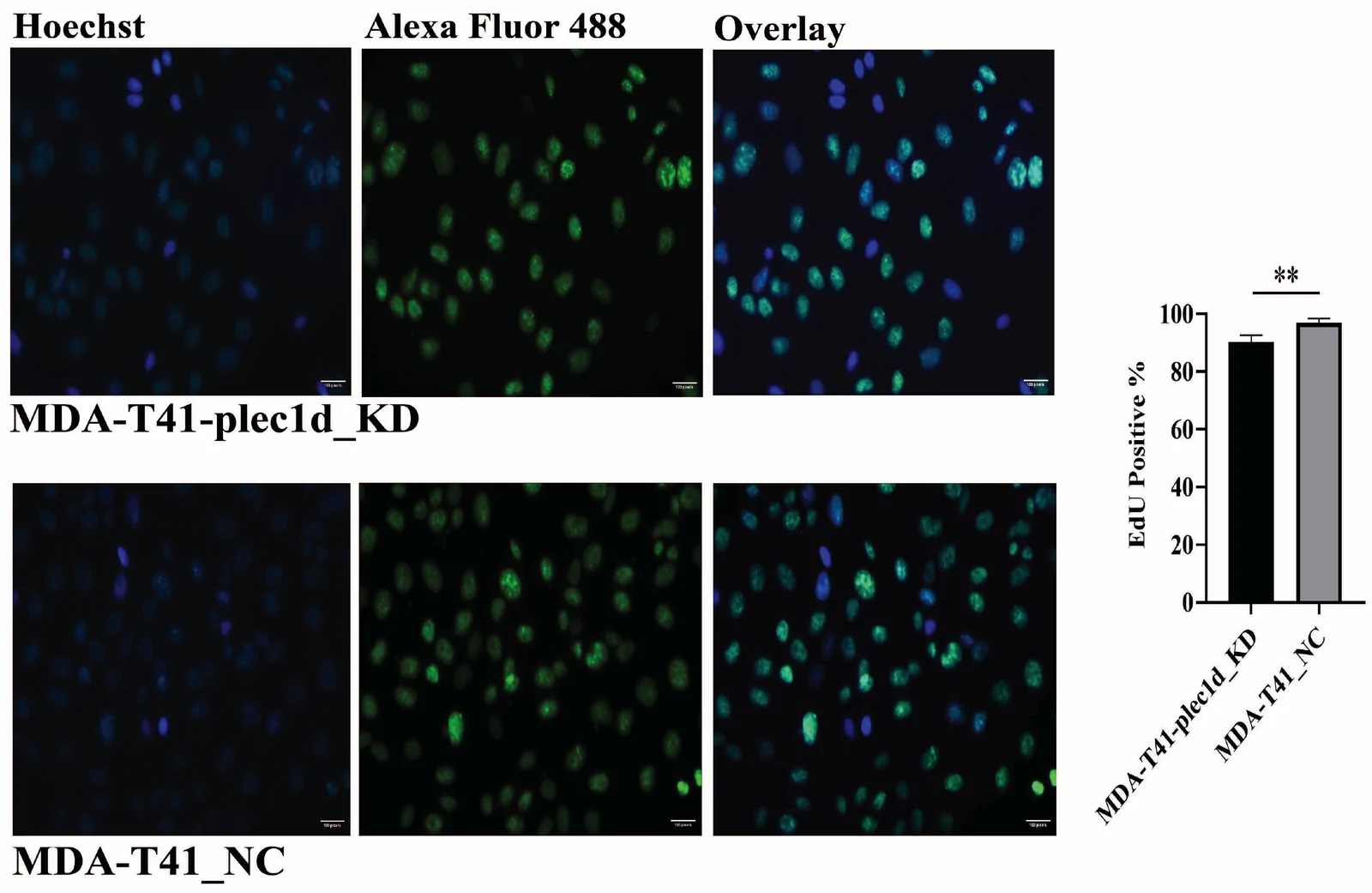
The effect of *PLEC 1d* KD on proliferation of MDA-T41 cells. Click-iT EdU assay demonstrated significantly diminished proliferation in MDA-T41-plec1d_KD cells compared to NC controls. *PLEC 1d* KD efficiency was 55.1% ± 2.3% (mean ± SD, *n* = 4). Blue: Hoechst (nuclei). Green: Alexa Fluor 488 (EdU). Objective magnification: 20×. Scale bar: 100 pixels (1 pixel = 0.3225 μm). ** *p* < 0.01.

The effect of *PLEC 1d* suppression on cell proliferation was subsequently investigated. Compared to the MDA-T41_NC group, the MDA-T41-plec1d_KD group showed a statistically significant 6.6% reduction in proliferation (*p* = 0.003) (Figure 3, Supplementary Figure S3).

**Figure 4:**
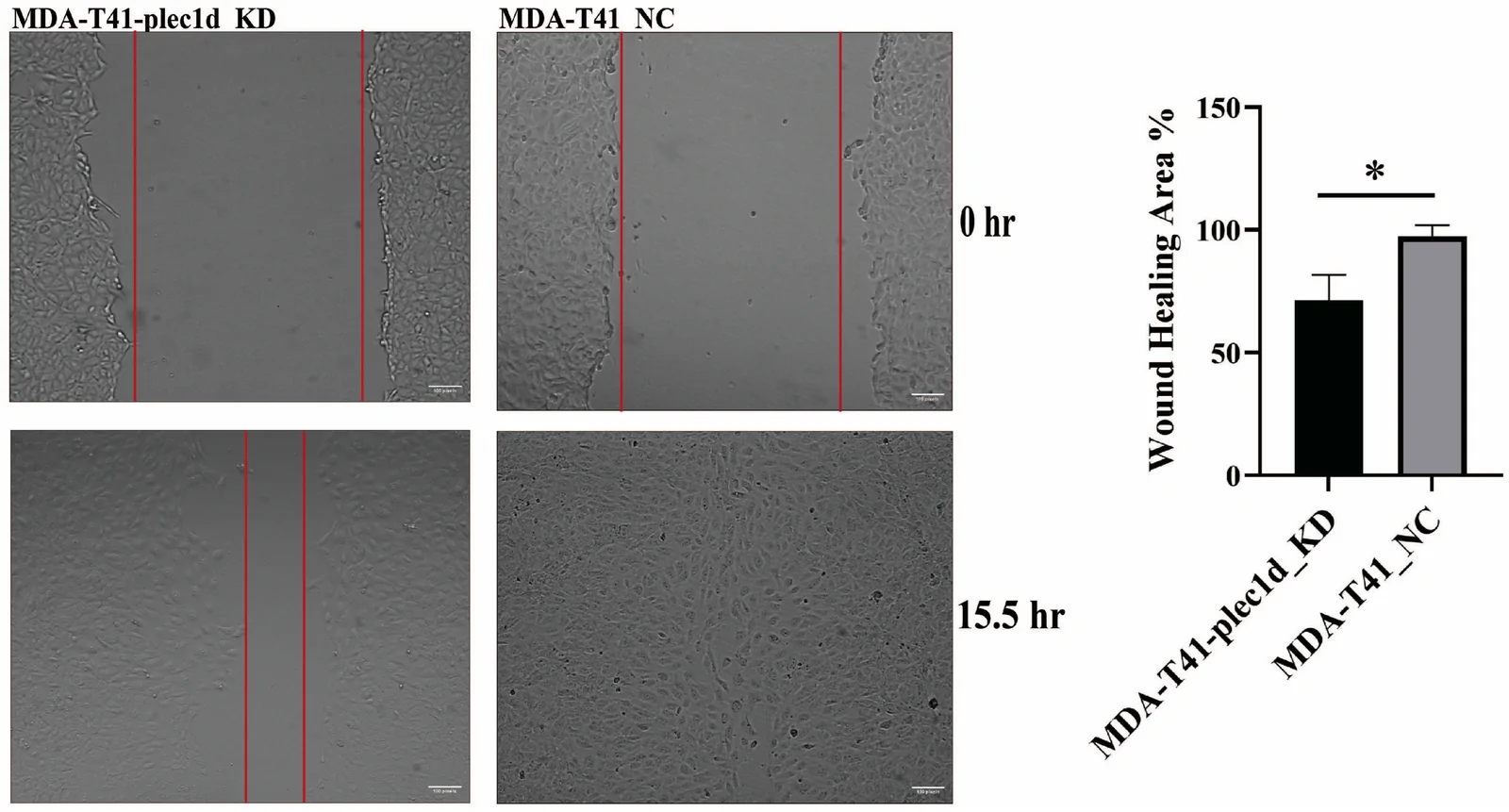
The effect of *PLEC 1d* KD on migration of MDA-T41 cells. Wound-healing assay showed significantly reduced migration in *PLEC 1d* KD MDA-T41 cells relative to NC. Mean knockdown efficiency: 50% ± 9.7% (mean ± SD, *n* = 3). Red lines show the wound edges. Objective magnification: 20×. Scale bar: 100 pixels (1 pixel = 0.3225 μm). * *p* < 0.05.

Wound healing assays showed that *PLEC 1d* KD significantly reduced cell migration by 26% in MDA-T41 cells relative to the NC group (*p* = 0.0163) (Figure 4, Supplementary Figure S4).

### 2.3. Transcriptome Data and a Potential Link Between PLEC 1d and PTC Metastasis

**Figure 5:**
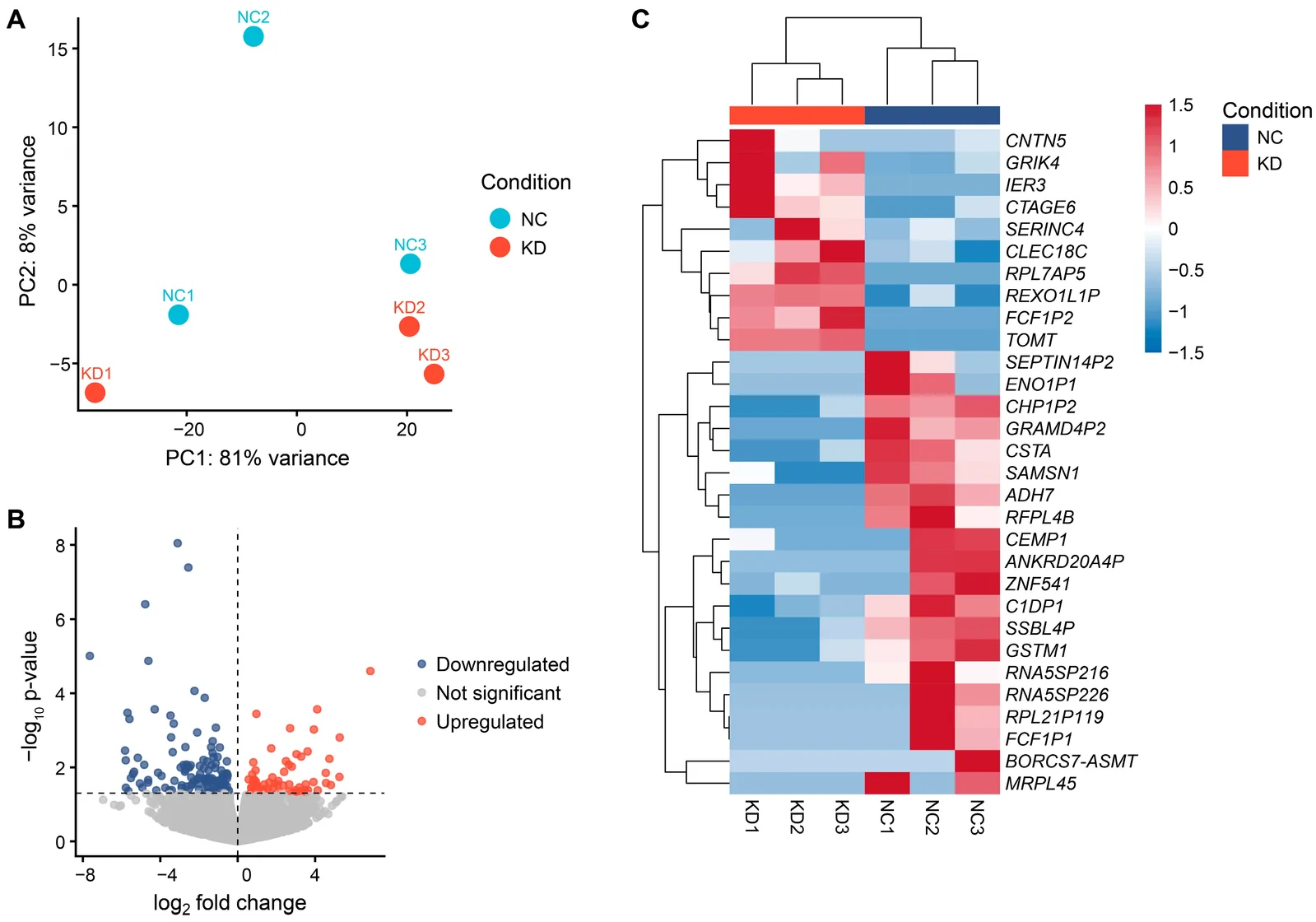
Transcriptomic profiling of *PLEC 1d* KD MDA-T41 cell line (KD) compared to NC. (**A**) PCA plot showing variance stabilizing transformation (vst)-normalized gene expression patterns of KD (red) and NC (blue) samples. (**B**) Volcano plot showing DEGs in KD relative to NC. (**C**) Heatmap showing hierarchical clustering of the top 30 DEGs based on vst-normalized expression levels in KD and NC samples.

Principal component analysis (PCA) was performed using variance stabilizing transformation (vst)-normalized expression data to evaluate the distribution of transcriptome profiles on a sample-by-sample basis from RNA-seq data (Figure 5A). PCA results showed that MDA-T41 *PLEC 1d* KD samples were clearly separated from MDA-T41 NC samples, whereas samples within the same group clustered closely together (Figure 5A).

Differential expression analysis identified 170 differentially expressed genes (DEGs) in the KD group compared to the NC group according to the *p*-value criterion, of which 112 were downregulated and 58 were upregulated (Figure 5B). The top 30 genes showing the highest expression change were visualized using a heatmap (Figure 5C). The KD group exhibited a greater number of significantly downregulated genes compared to upregulated ones, suggesting that *PLEC 1d* KD mainly acts to suppress gene expression in MDA-T41 PTC cells.

**Figure 6:**
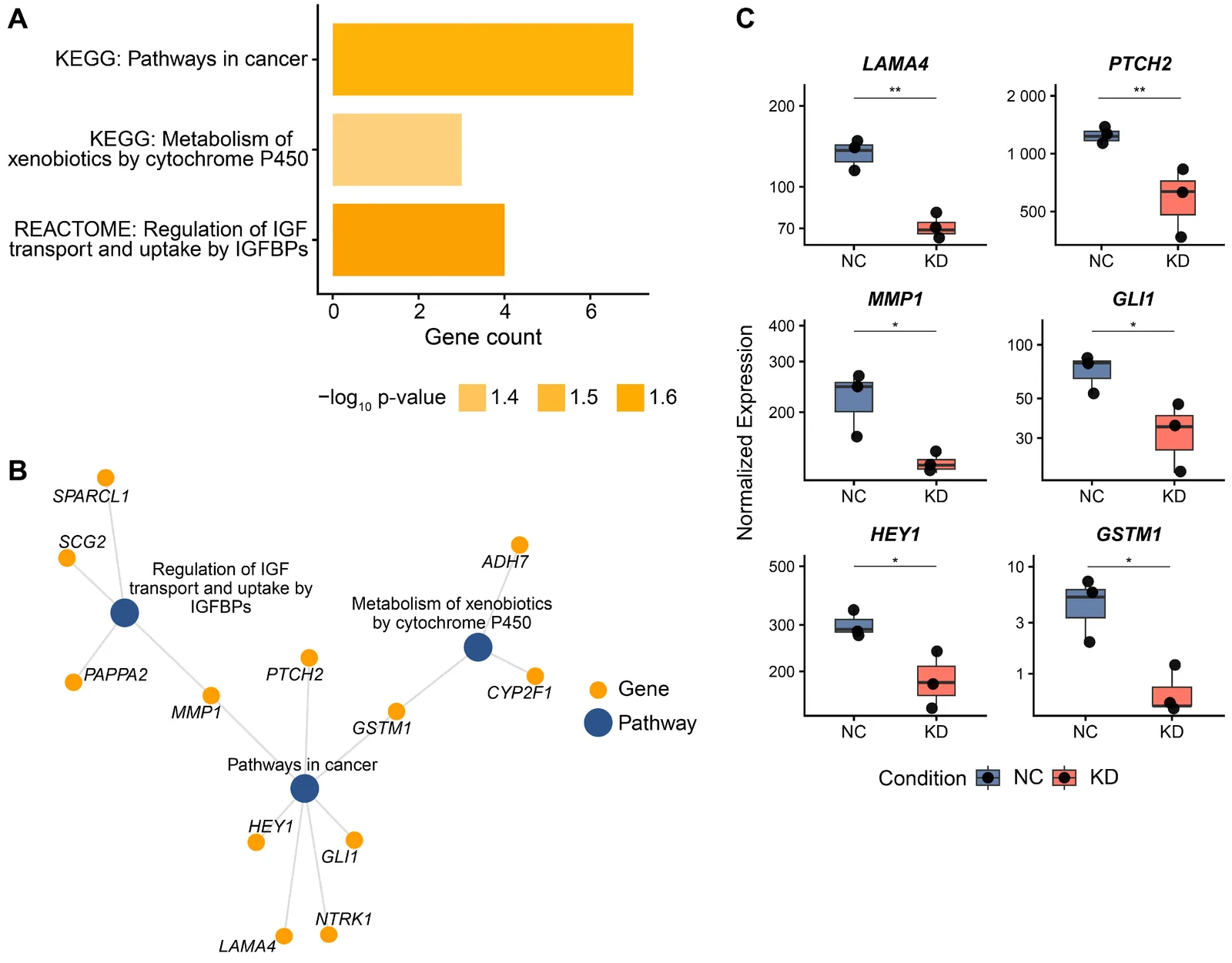
Pathway enrichment analyses for downregulated genes. (**A**) Bar plot showing statistically significant pathways enriched among downregulated genes. (**B**) Network representation of downregulated genes associated with significantly enriched pathways. (**C**) Box plots showing vst-normalized expression levels of DEGs involved in the “Pathways in cancer” pathway in the KD and NC groups. Significance levels based on DESeq2 *p* values: * *p* < 0.05, ** *p* < 0.01.

Additionally, pathway enrichment analysis was performed to identify molecular pathways involving downregulated and upregulated genes. Downregulated genes showed significant enrichment in three pathways, with the *p*-value < 0.05 as the cut-off criterion: “Pathways in cancer”, “Metabolism of xenobiotics by cytochrome P450”, and “Regulation of insulin-like growth factor (IGF) transport and uptake by insulin-like growth factor-binding proteins (IGFBPs)” (Figure 6A). The connections between downregulated genes and significantly enriched pathways were visualized using network analysis (Figure 6B).

**Figure 7:**
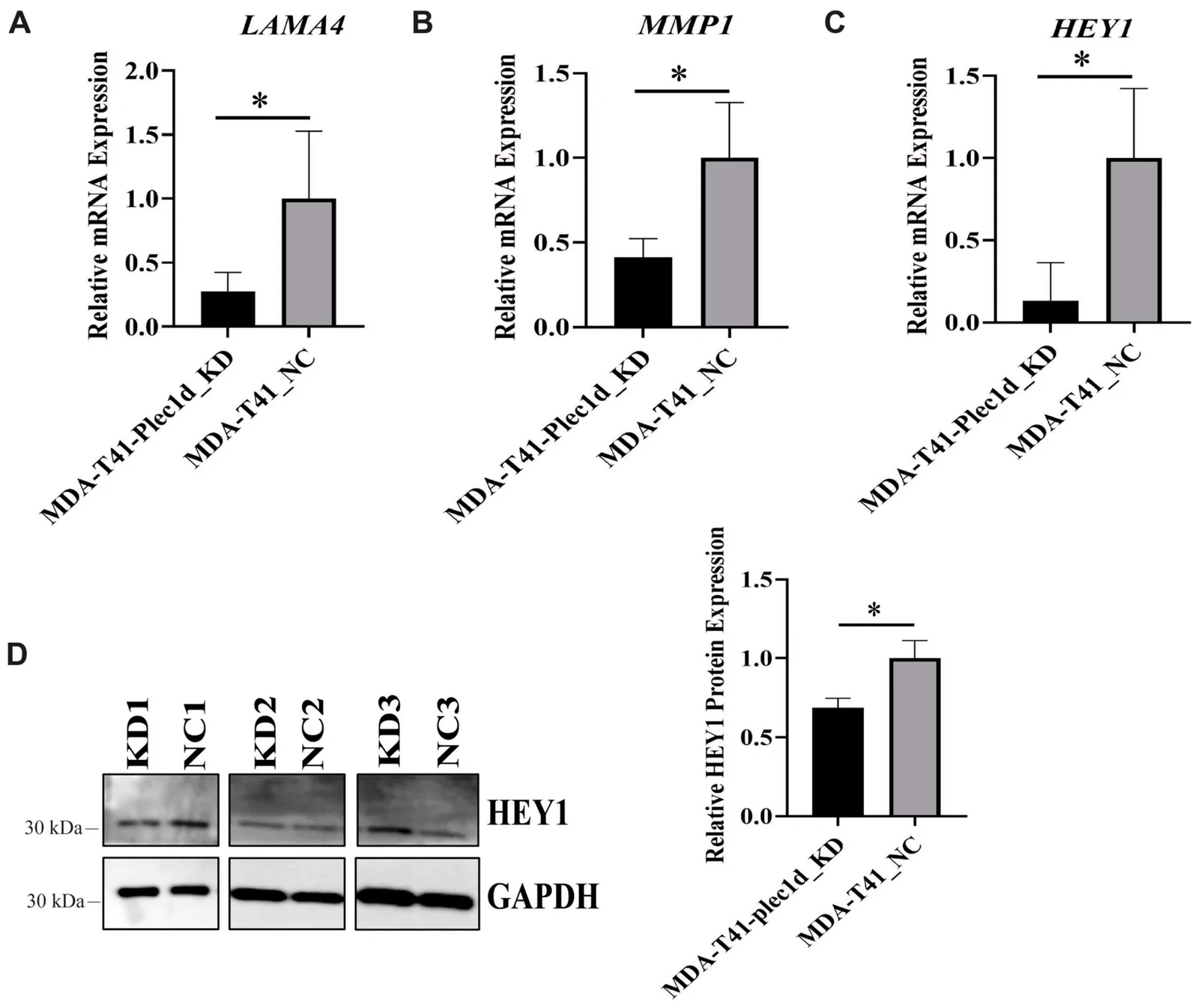
Expression of *LAMA4*, *MMP1*, and HEY1 in *PLEC 1d* KD MDA-T41 cells compared to NC. (**A**) *LAMA4* mRNA expression level. (**B**) *MMP1* mRNA expression level. (**C**) *HEY1* mRNA expression level. (**D**) Western blot analysis and relative HEY1 protein (~33 kDa) expression level in *PLEC 1d KD* MDA-T41 cells compared to NC. GAPDH (37 kDa) is the loading control. * *p* < 0.05.

The expression levels of differentially expressed genes associated with the ‘Pathways in cancer’ were also examined (Figure 6C). All analyzed genes exhibited reduced expression in the KD group compared to the NC group, with significant decreases observed for Laminin Subunit Alpha 4 (*LAMA4*), Patched 2 (*PTCH2*), Matrix Metallopeptidase 1 (*MMP1*), Hes Related Family BHLH Transcription Factor with YRPW Motif 1 (*HEY1*), and GLI family zinc finger 1 (*GLI1*) (Figure 6C). Among these, *LAMA4*, *MMP1*, and *HEY1*, each implicated in cancer development and progression, were selected as target genes to validate the transcriptomic findings by assessing their expression levels using RT-qPCR. (Figure 7A–C). Thus, consistent with the RNA-seq results, *LAMA4*, *MMP1*, and *HEY1* were significantly downregulated after *PLEC 1d* KD in MDA-T41 cells relative to MDA-T41_NC (*p* = 0.043, *p* = 0.036, and *p* = 0.023, respectively) (Figure 7A–C). In addition, LAMA4 and MMP1 are secreted proteins and were not detected in total cell lysates in this study. Therefore, only HEY1 protein expression was assessed in *PLEC 1d* KD MDA-T41 cells compared to MDA-T41_NC, confirming that HEY1 expression decreased after *PLEC 1d* suppression (Figure 7D).

### 2.4. mRNA and Protein Expression Profiles of Three Candidate Genes in PTC Cell Lines

**Figure 8:**
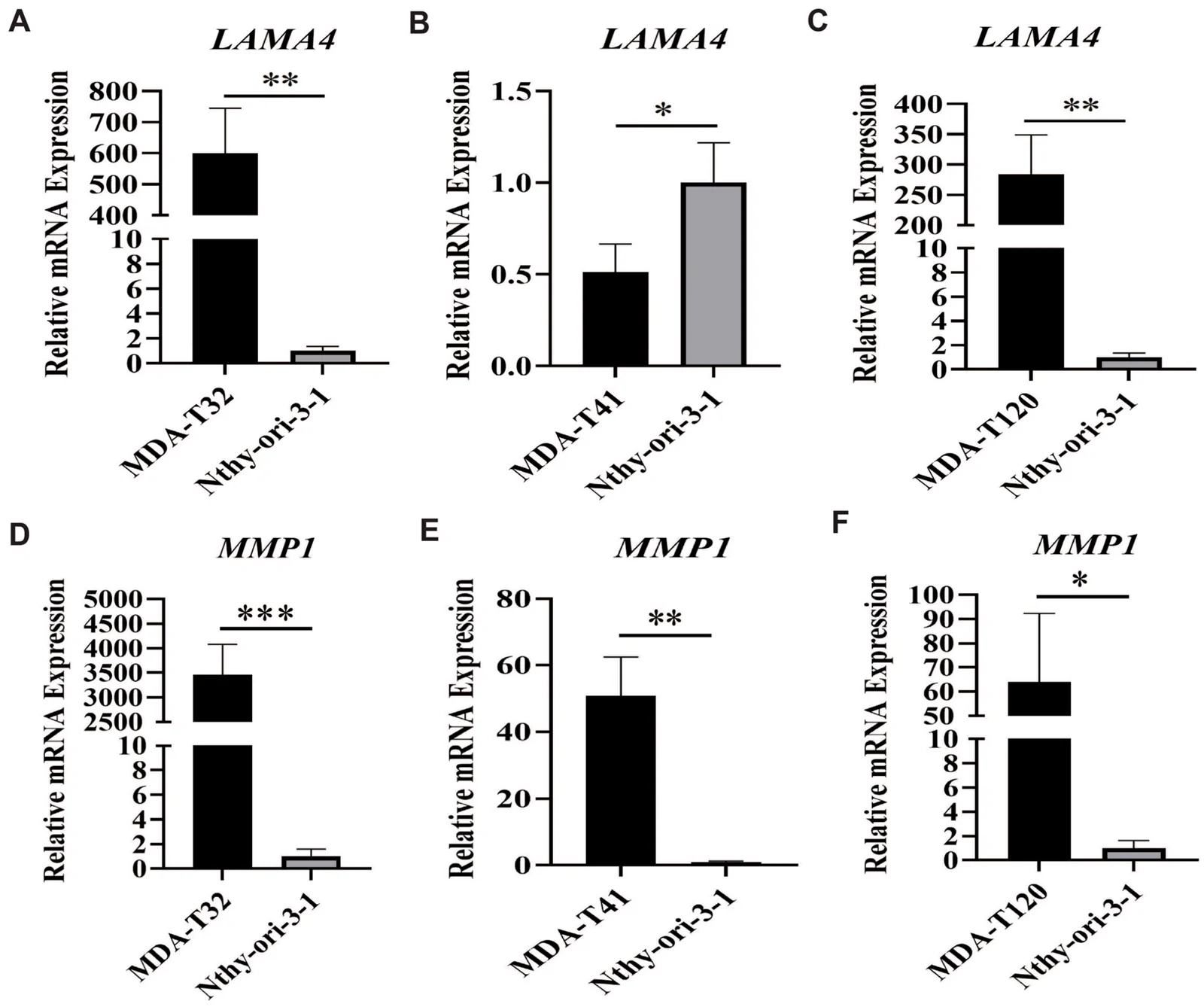
mRNA expression levels of *LAMA4* and *MMP1* in PTC cell lines. *LAMA4* mRNA expression in MDA-T32 (**A**), MDA-T41 (**B**), and MDA-T120 (**C**). *MMP1* mRNA expression in MDA-T32 (**D**), MDA-T41 (**E**), and MDA-T120 (**F**). * *p* < 0.05, ** *p* < 0.01, *** *p* < 0.001.

**Figure 9:**
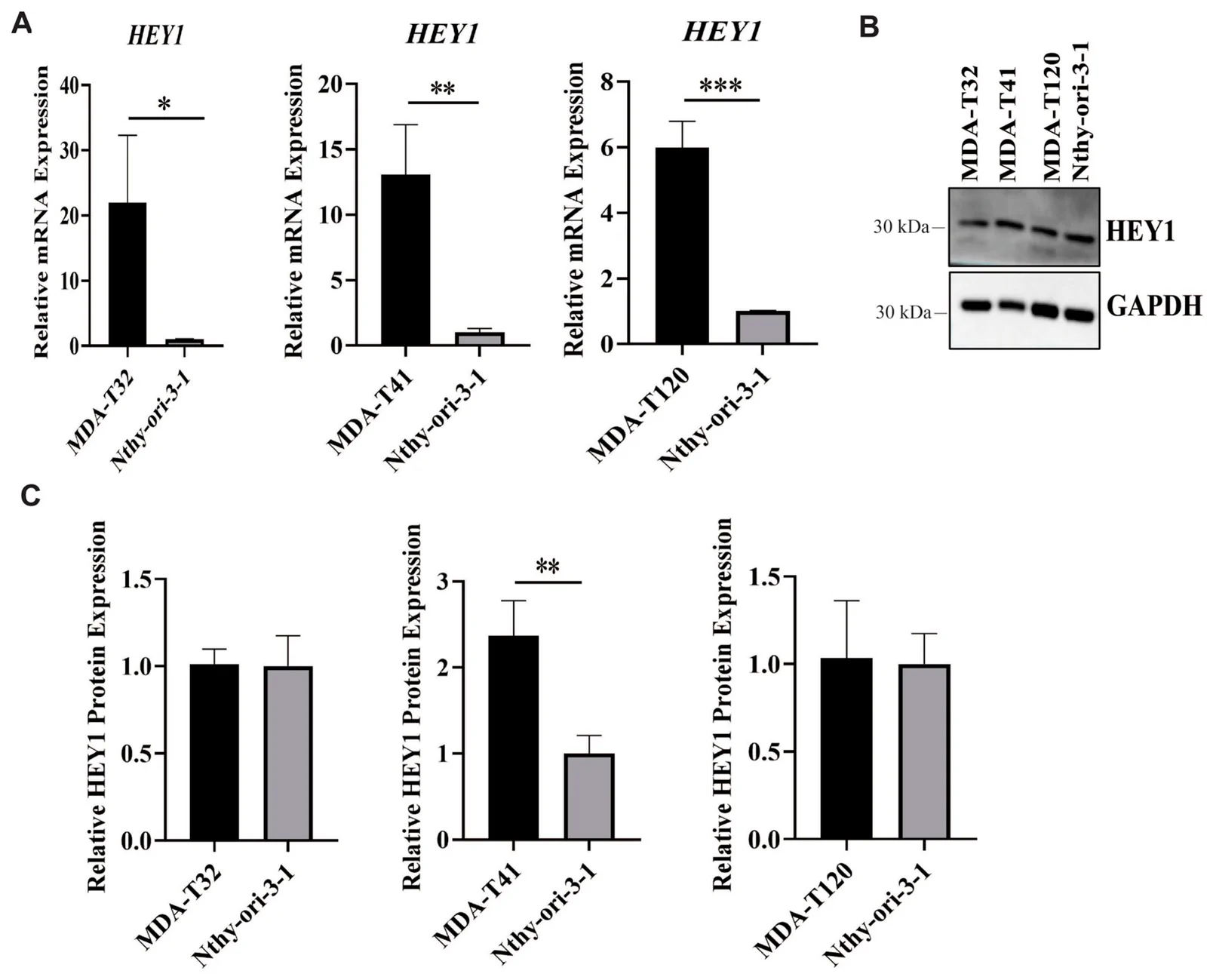
HEY1 expression in PTC cell lines at both the transcript and protein levels. (**A**) RT-qPCR analysis of *HEY1* mRNA expression in MDA-T32, MDA-T41, and MDA-T120 cells. (**B**) Western blot showing HEY1 protein (~33 kDa) expression; GAPDH (37 kDa) was used as a loading control. (**C**) Densitometric quantification of HEY1 protein expression relative to the normal thyroid cell line Nthy-ori-3-1. * *p* < 0.05, ** *p* < 0.01, *** *p* < 0.001.

RT-qPCR validation revealed the differential regulation of the target genes among PTC cell lines. Accordingly, *LAMA4* expression was significantly elevated in MDA-T32 and MDA-T120 cells but decreased in MDA-T41 cells (Figure 8A–C). In contrast, *MMP1* mRNA levels were significantly upregulated in all three cell lines (Figure 8D–F). Similarly, *HEY1* expression increased significantly across all three PTC cell lines (Figure 9A).

Due to the secreted nature of LAMA4 and MMP1, only HEY1 protein expression was assessed in total cell lysates from the three PTC cell lines (Figure 9B). The results demonstrated a significant upregulation of HEY1 protein in MDA-T41 cells (*p* = 0.0058), while no significant changes were observed in MDA-T32 (*p* = 0.74) or MDA-T120 (*p* = 0.92) cells (Figure 9B,C, Supplementary Figure S5).

## 3. Discussion

The present study investigated the role of the *PLEC 1d* isoform in regulating migratory capacity in PTC. Of the three PTC cell lines analyzed, only MDA-T41 cells showed elevated *PLEC 1d* mRNA expression and increased total plectin protein levels. Functional assays indicated that *PLEC 1d* KD resulted in a modest decrease in cell proliferation and a pronounced inhibition of cell migration. The transcriptomic profiling of *PLEC 1d* KD MDA-T41 cells revealed coordinated alterations in genes associated with invasion and migration. Notably, HEY1 was identified as a potential downstream effector regulated by the *PLEC 1d* isoform.

A previous pan-cancer analysis revealed that *PLEC 1d* is significantly upregulated in THCA and exhibits high diagnostic performance (AUC = 0.809), suggesting a potential role in THCA pathogenesis [9]. In this study, *PLEC 1d* mRNA expression was not detected in the MDA-T32, MDA-T120, and healthy control Nthy-ori-3-1 cell lines. In contrast, a marked increase in expression was observed in the MDA-T41 cell line. Based on these results, the MDA-T32, MDA-T41, and MDA-T120 cell lines were further analyzed regarding their genetic backgrounds and metastatic characteristics. Accordingly, the MDA-T32 cell line was derived from a primary PTC in a 74-year-old male patient exhibiting extrathyroidal extension, cervical lymph node metastasis, and lymphovascular invasion [28]. This cell line harbors BRAF V600E and TERT promoter −124C>T mutations. The MDA-T41 cell line was established from metastatic lymph nodes of a 74-year-old male patient with recurrent PTC, characterized by extracapsular extension and lymphovascular invasion [28], and carries only the BRAF V600E mutation. The MDA-T120 cell line derived from a metastatic lymph node lesion of a 72-year-old female PTC patient who exhibited extracapsular extension and lymphovascular invasion [28]. This line harbors BRAF V600E, TP53 R280T, and TERT promoter −124C>T mutations.

Although both MDA-T41 and MDA-T120 cell lines originated from metastatic lesions, *PLEC 1d* overexpression was detected exclusively in MDA-T41 cells. These results indicate that *PLEC 1d* is unlikely to serve as a universal marker of metastasis and may instead be linked to a distinct aggressive or cytoskeleton-remodeling phenotype in PTC. Additionally, a previous study demonstrated that the lncRNA HOTAIR is highly expressed in MDA-T32 cells but markedly reduced in MDA-T41 [29]. Collectively, all these findings underscore the substantial molecular heterogeneity underlying PTC.

Consistent with the mRNA expression data, Western blot analysis demonstrated a significant increase in total plectin protein expression in the MDA-T41 cell line compared with the healthy cell line, Nthy-ori-3-1. The concordance between elevated *PLEC 1d* mRNA levels and increased plectin protein expression in MDA-T41 cells indicates that upregulation of the plectin 1d isoform may substantially contribute to the overall increase in plectin protein levels in these cells. In contrast, no statistically significant difference in total plectin protein expression was detected in the MDA-T32 cell line relative to Nthy-ori-3-1, and no statistically significant change was observed in the MDA-T120 cell line. These results align with the corresponding *PLEC 1d* mRNA expression patterns, further supporting the isoform-specific contribution of plectin 1d to the overall plectin expression profile.

Functional assays demonstrated that partial KD of *PLEC 1d* expression (≥50%) in MDA-T41 cells resulted in a statistically significant reduction in cell proliferation, as measured by EdU incorporation analysis. The phenotypic effect on proliferation was relatively modest. This result suggest that *PLEC 1d* is not a primary driver of cell proliferation but may contribute to subtle modulation of cell cycle progression. Thus, reduction in *PLEC 1d* expression appears to slow cell cycle kinetics rather than strongly suppress proliferative capacity.

In contrast to the limited effect on proliferation, *PLEC 1d* KD resulted in a pronounced decrease in cell migration capacity, indicating a substantial role for this isoform in regulating the migratory behavior of MDA-T41 PTC cells. Given the established function of plectin as a cytoskeletal crosslinking protein that integrates intermediate filaments with other cytoskeletal components, these findings suggest that plectin 1d may contribute to cytoskeletal organization and cell motility in PTC cells. Consequently, the elevated expression of *PLEC 1d* observed in the MDA-T41 cell line may facilitate enhanced migratory capacity, suggesting a potential role in promoting an aggressive cellular phenotype that could be relevant to metastatic behavior in PTC.

Additionally, transcriptome analysis with the *PLEC 1d* KD MDA-T41 PTC cell line revealed significantly downregulated genes which were potentially associated with cell invasion and migration. Among these, *LAMA4*, a major basement membrane component of the laminin family, is essential for cell survival, proliferation, and migration in various cell types [30,31]. Elevated *LAMA4* expression correlates with poor survival, metastasis, and increased cell migration in renal cell carcinoma [32]. Further, increased *LAMA4* expression has been associated with enhanced cell invasion in breast cancer [33]. In the present study, basal *LAMA4* expression was already lower in untreated MDA-T41 cells compared to normal thyroid Nthy-ori-3-1 cells. Notably, *PLEC 1d* KD further significantly reduced *LAMA4* expression in MDA-T41 cells relative to NC cells, suggesting that *PLEC 1d* may act as a positive regulator of *LAMA4*, at least in this cellular context. While our data establish a functional correlation between *PLEC 1d* and *LAMA4* expression, the underlying molecular mechanisms remain to be elucidated. One possibility, based on the observation that basal *LAMA4* transcript levels are already reduced in MDA-T41 cells, is that *PLEC 1d* might operate through post-transcriptional or translational pathways. For instance, it could hypothetically enhance *LAMA4* mRNA stability via interactions with RNA-binding proteins or cytoskeletal elements. Alternatively, it might facilitate protein synthesis or prevent proteasomal degradation of laminin subunits. However, we emphasise that these proposed mechanisms are currently speculative and are not directly tested by our experimental data. The further reduction in *LAMA4* expression following *PLEC 1d* KD, even when basal levels are already low, raises the intriguing possibility that *PLEC 1d* may influence LAMA4 at more than one level; nevertheless, this hypothesis requires rigorous validation in future studies using approaches such as RNA stability assays, polysome profiling, or ubiquitination analyses. In contrast, increased *LAMA4* expression observed in other PTC cell lines such as MDA-T32 and T120 indicates a possible cell line-specific regulation. These differences may reflect variations in molecular subtype, invasive potential, extracellular matrix remodelling capacity, or basal transcriptional programmes among PTC models. Collectively, these results highlight that the relationship between *PLEC 1d* and LAMA4 is cell line-specific and may vary across distinct genetic backgrounds or subtypes of PTC.

MMP1 is a zinc-dependent endopeptidase belonging to the matrix metalloproteinase (MMP) family and has been implicated in inflammation, malignancy, and cardiovascular and renal diseases [34]. Among MMP family members, MMP1, MMP2, and MMP9 are particularly associated with tumor progression, extracellular matrix remodeling, metastasis, and local invasion in multiple cancer types [35]. Previous studies have also demonstrated that suppression of MMP1 can inhibit colorectal cancer progression, supporting its pro-tumorigenic role [36]. In THCA, elevated *MMP1* expression has been reported in invasive PTC and anaplastic thyroid carcinoma (ATC) [37,38]. Although the role of MMP1 in PTC and poorly differentiated/ATC (PDTC/ATC) has been investigated in several studies, its association with tumor differentiation status and prognosis remains incompletely understood. Some reports have shown that increased *MMP1* expression correlates with advanced clinical stage and aggressive tumor behavior in PTC [39,40,41]. However, the prognostic significance of *MMP1* expression across different thyroid cancer subtypes remains uncertain. In the present study, *MMP1* mRNA expression was significantly decreased in *PLEC 1d* KD MDA-T41 PTC cells compared with NC cells, suggesting a potential relationship between *PLEC 1d* expression and MMP1-associated invasive pathways. Nevertheless, *MMP1* expression was elevated across all examined PTC cell lines (MDA-T32, MDA-T41, and MDA-T120). This widespread upregulation indicates that MMP1 may represent a general feature of PTC biology rather than a marker capable of discriminating metastatic aggressiveness among these cell lines. Collectively, these findings suggest that while MMP1 may contribute to the invasive phenotype of THCA, its expression alone may be insufficient to distinguish differences in metastatic potential between PTC models.

Another target gene, *HEY1*, belongs to the basic helix loop helix (bHLH) family of transcription factors. Members of this family selectively bind to specific DNA sequences to regulate target gene expression, thereby controlling the intensity, timing, and spatial distribution of gene expression [42]. HEY1 is also recognized as an effector of the Notch signaling pathway [43,44]. Multiple studies have demonstrated that HEY1 plays a significant role in tumor progression across various cancer types. For example, Pu et al. found that the upregulation of *HEY1* in melanoma cells enhances invasion and metastasis by modulating the GRB2/PI3K/AKT signaling pathway [45]. In human osteosarcoma (OS) cells, *HEY1* overexpression promotes invasiveness and facilitates lung metastasis by increasing MMP9 expression [46]. HEY1 also serves as a critical regulator of epithelial–mesenchymal transition (EMT). Xu et al. demonstrated that HEY1 expression is regulated by the transforming growth factor-β (TGF-β) signaling pathway, underscoring its role in EMT-mediated tumor progression [47]. Furthermore, it was reported that activated Notch1 or HEY1 expression may serve as prognostic markers in PTC [48]. Activated Notch1 expression was lower in cases with lymph node metastasis and higher in those without metastasis. HEY1 expression correlated with activated Notch1 expression and was reduced in cases with extrathyroidal extension compared to those without [48]. In another study, co-expression of the BRAF V600E mutation and overexpression of the Notch intracellular domain resulted in significantly larger thyroid tumors in mice, promoted a more aggressive carcinoma phenotype, and reduced overall survival [49]. PTC cell lines with the BRAF V600E mutation were resistant to pharmacological inhibition of the Notch pathway. Inhibition of MEK1/2 had a greater effect on Hes1/Hey1 transcription than Notch inhibition in BRAF V600E mutant cell lines. HEY1 expression was also elevated in these cell lines due to the activation of the MAP kinase pathway [49]. Despite some conflicting findings, current evidence indicates that HEY1 expression should be considered in the pathogenesis of PTC. Thus, our study demonstrated that HEY1 is downregulated by the decreased expression of *PLEC 1d* both at the mRNA and protein levels in MDA-T41 cells. However, HEY1 mRNA expression is increased across all PTC cell lines but it is only significantly upregulated in MDA-T41 cell line at the protein level. These findings suggest that post-transcriptional or translational mechanisms such as differential mRNA stability, microRNA-mediated repression, or altered proteasomal degradation may govern HEY1 protein expression in a cell line-specific manner. *PLEC 1d* may act at multiple levels to influence HEY1, and its effect on aggressive behavior likely depends on the post-transcriptional landscape of each cellular context.

While this study offers novel insights into the potential role of *PLEC 1d* in PTC progression, several limitations should be acknowledged. Thus, functional analyses were primarily conducted in a single PTC cell line (MDA-T41), which may restrict the generalizability of the findings given the molecular heterogeneity of PTC. Although MDA-T32 and MDA-T120 cells were included for comparative expression analyses, further functional validation across multiple PTC models would enhance the robustness of the observed associations. The KD efficiency of *PLEC 1d* was partial, likely due to the isoform-specific targeting strategy, and residual expression may have diminished the phenotypic effects. Additionally, transcriptomic findings were validated only for selected candidate genes, and the molecular mechanisms linking *PLEC 1d* to *HEY1*, *MMP1*, and *LAMA4* associated pathways remain incompletely understood. It should be noted that LAMA4 and MMP1 were validated only at the transcript level, as protein-level detection by Western blot was unsuccessful in our system. More sensitive approaches will be needed for protein confirmation in future studies. The lack of in vivo metastasis models and patient-derived clinical validation further limits the translational relevance of these findings. Future studies incorporating broader functional assays, animal models, and clinical cohorts are warranted to clarify the role of *PLEC 1d* in PTC progression and metastasis.

In conclusion, this study demonstrates that the *PLEC 1d* isoform may contribute to the aggressive phenotype of PTC by enhancing migratory capacity and modulating transcriptional programs associated with invasion. Among the PTC cell lines analyzed, *PLEC 1d* overexpression was observed exclusively in the metastatic MDA-T41 model, underscoring the molecular heterogeneity of PTC and indicating that *PLEC 1d* is associated with a distinct subtype-specific phenotype rather than functioning as a universal metastatic marker. Functional suppression of *PLEC 1d* led to reduced cell migration and modest inhibition of proliferation, accompanied by coordinated downregulation of genes involved in extracellular matrix remodeling and tumor progression, such as *LAMA4*, *MMP1* and *HEY1*. Notably, HEY1 emerged as a potential downstream effector of *PLEC 1d*, raising the possibility that this axis may contribute to pathways relevant to tumor progression and metastatic competence. Collectively, these findings indicate that *PLEC 1d* may serve as a key regulator of cytoskeletal organization and could influence processes associated with metastatic potential in a subset of PTCs. Further mechanistic and clinical studies are warranted to determine whether *PLEC 1d* might serve as a biomarker or a personalized therapeutic target in aggressive PTC.

## 4. Materials and Methods

### 4.1. Cell Culture

The PTC cell lines MDA-T32 (CRL-3351), MDA-T41, and MDA-T120 (CRL-3355), as well as the healthy human thyroid follicular epithelial cell line Nthy-ori-3-1 (Lot: 09C008), were utilized in this study. MDA-T32 and MDA-T120 were obtained from the *American Type Culture Collection* (ATCC), MDA-T41 was provided by Dr. Alessia Ciarrocchi (Laboratory of Translational Research, Reggio Emilia, Italy), and Nthy-ori-3-1 was purchased from Sigma. PTC cell lines MDA-T32, MDA-T41, and MDA-T120 were cultured in RPMI 1640 (ATCC) supplemented with 10% fetal bovine serum (FBS; Gibco, Paisley, UK) and 1% penicillin/streptomycin (Gibco). The healthy control Nthy-ori-3-1 cell line was cultured in RPMI 1640 (ATCC) supplemented with 10% FBS (Gibco), 1% L-glutamine (Biowest, Nuaillé, France), and 1% penicillin/streptomycin (Gibco).

### 4.2. RNA Extraction and cDNA Synthesis

Total RNA was extracted from MDA-T32, MDA-T41, MDA-T120, and Nthy-ori 3-1 cells using the PureLink RNA kit (Thermo Fisher, Carlsbad, CA, USA) according to the manufacturer’s protocol. RNA concentration was determined with the RNA Assay Broad Range kit on a Qubit 4 instrument. Complementary DNA (cDNA) was synthesized from 1 µg of RNA using the High-Capacity cDNA Reverse Transcription kit (Thermo Fisher, Vilnius, Lithuania) according to the manufacturer’s instructions.

### 4.3. Real Time Polymerase Chain Reaction (RT-PCR)

*PLEC 1d* mRNA expression in PTC cell lines was evaluated using reverse transcription polymerase chain reaction (RT-PCR). After a 1:5 dilution of cDNA, amplification was conducted using the Ampigene qPCR Green Mix with the Hi-ROX kit (ENZO, Framingdale, NY, USA). Glyceraldehyde 3-phosphate dehydrogenase (*GAPDH*) served as the loading control. *GAPDH* primers used in this study, Forward (F): 5′-CCAGAACATCATCCCTGCCT- 3′, Reverse (R): 5′-CCTGCTTCACCACCTTCTTG- 3′. RT-PCR products were visualized by electrophoresis on a 3% agarose gel.

### 4.4. Transfection

MDA-T41 cells were seeded at a density of 2 × 10^5^ cells per well in 6-well plates and incubated at 37 °C with 5% CO_2_ for 24 h. The CRISPR-Cas9 system was employed to silence the *PLEC 1d* isoform. Single-guide RNA (sgRNA) sequences were designed using the Custom Alt-R CRISPR-Cas9 gRNA tool by targeting the unique exon 1d sequence, which is exclusive to the *PLEC 1d* and absent in other plectin isoforms. Two distinct sgRNAs targeting *PLEC 1d* (NM_201381.3) were generated and employed them as a pool: sgRNA_1 (ACGAUCUUCAUCGGGGACGG) and sgRNA_2 (GGCACGAUCUUCAUCGGGGA). In addition, an sgRNA negative control (sgRNA_NC, TrueGuide Negative Control) was included. Transfection was performed according to the Invitrogen TrueGuide Synthetic gRNA protocol, and cells were incubated for 48 h post-transfection. A mixed population of edited cells was used, and only transfections achieving ≥50% reduction in *PLEC 1d* mRNA expression were selected for functional assays. This study repeated at least three times for all experiments.

### 4.5. Realtime Quantitative Polymerase Chain Reaction (RT-qPCR)

The study aimed to determine the efficiency of *PLEC 1d* KD and to validate RNA sequencing data. Amplifications were performed using the Ampigene qPCR Green Mix with Hi-ROX kit (ENZO, Framingdale, NY, USA). When this kit was no longer commercially available, PowerUp SYBR Green Master Mix (Thermo Fisher, Vilnius, Lithuania) was used as a replacement.

mRNA expression levels were normalized to *GAPDH* and *ACTB* as reference genes. The Nthy-ori-3-1 cell line served as the calibrator (healthy control) for the PTC cell lines, while MDA-T41_Negative Control (MDA-T41_NC) served as the calibrator for *PLEC 1d*-suppressed MDA-T41 (MDA-T41-plec1d_KD) cells. Relative quantification was calculated using the 2^−ΔΔ*Ct*^ method. Statistical significance was assessed using one or two-tailed Student *t*-tests, with *p* < 0.05 considered significant. Primer sequences are provided in the Supplementary Table S1.

### 4.6. Protein Extraction and Western Blot

Total protein was extracted from the cells using Radioimmunoprecipitation Assay (RIPA) lysis buffer (Thermo Fisher, Rockford, IL, USA) supplemented with 1% protease inhibitor (Halt Protease Inhibitor Cocktail, Thermo Fisher, Rockford, IL, USA). Lysates containing 50 μg of protein were treated with 4× NuPAGE LDS Sample Buffer and 10× NuPAGE Sample Reducing Agent, then denatured at 70 °C for 10 min. Protein samples were loaded onto a 7% NuPAGE Tris-Acetate gel. A 20× Tris-Acetate SDS running buffer (Novex, Carlsbad, CA, USA) supplemented with 500 µL of NuPAGE Antioxidant was used. Following electrophoresis, the gel was transferred to a PVDF membrane using NuPage Transfer Buffer (Invitrogen, Carlsbad, CA, USA). The transfer was performed at 250 mA on ice for 3 h. GAPDH and ATP1A1 were used as loading controls. Since the extended electrophoresis necessary for resolving plectin (~500 kDa) resulted in the complete loss of the GAPDH signal, ATP1A1 (100–110 kDa) was employed as the loading control for plectin expression analysis. However, for other protein expression assays, GAPDH was employed as the loading control. As a specific antibody against plectin 1d is not commercially available, total plectin protein levels were assessed using a plectin antibody (ab32528, Abcam, Cambridge, UK) at a 1:5000 dilution. The other primary antibodies used included GAPDH (MA5-35235, Invitrogen, Rockford, IL, USA) at 1:10,000, ATP1A1 (Proteintech, Wuhan, China) at 1:4000, and HEY1 (STJ193066, London, UK) at 1:1000. The secondary antibody employed was S0001 (Affinity, Jiangsu, China). Protein bands were detected using the SuperSignal West Femto Maximum Sensitivity Substrate Kit (Thermo Fisher, Rockford, IL, USA). HiMark Prestained Protein Marker (Thermo Fisher, Carlsbad, CA, USA) or PrimeStep Prestained Broad Range Protein Ladder (BioLegend, 773302, San Diego, CA, USA) was selected based on the specific protein analyzed in this study. Densitometric analysis of the protein bands was conducted using ImageJ 1.54g, and the values were normalised to the corresponding loading controls.

### 4.7. Cell Proliferation Assay

After *PLEC 1d* expression was reduced by at least 50% in MDA-T41 cells, 2 × 10^5^ cells/mL from each group (MDA-T41_KD and MDA-T41_NC) were seeded into 35 mm Petri flasks containing sterile coverslips. Cell proliferation was evaluated using the Click-iT EdU Imaging kit, Alexa Fluor 488 (C10337, Thermo Fisher, Eugene, OR, USA). In this study, cells were incubated with 20 µM 5-ethynyl-2′-deoxyuridine (EdU) for 24 h. The manufacturer’s protocol was followed, and cells were visualized using a Zeiss Axio Vert. A1 fluorescence inverted microscope. Statistical significance was determined using a two-tailed Student *t*-test.

### 4.8. Wound Healing Assay

A total of 48 h after transfection in MDA-T41 cells, 2 × 10^5^ cells/mL from each group (MDA-T41_KD and MDA-T41_NC) were seeded into 24-well plates to confluence. The next day, cells were treated with 10 µg/mL Mitomycin (Serva, Heidelberg, Germany) to inhibit proliferation and incubated for 2 h at 37 °C in a 5% CO2 incubator. After incubation, a wound was generated using a 200 µL pipette tip. Cells were washed twice with 1× PBS, and images were captured at 0 h. Cells were subsequently examined and photographed 15 h and 30 min later using Zeiss Axio Vert. A1 fluorescence inverted microscope. Image analysis was conducted using the ImageJ Wound Healing Size Tool (https://github.com/AlejandraArnedo/Wound-healing-size-tool/wiki, accessed on 10 May 2026). A two-tailed Student *t*-test was used to assess statistical significance.

### 4.9. RNA Sequencing

This study used KD1, KD2, and KD3 MDA-T41 cells (with at least 50% suppression of *PLEC 1d*) and NC1, NC2, and NC3 MDA-T41 cells as negative controls. Total RNA was extracted from each group using the PureLink RNA kit (Thermo Fisher, Carlsbad, CA, USA) following the manufacturer’s protocol. RNA sample quality was assessed using a Nanodrop and an RNA Quantification HS kit on a Thermo Fisher Qubit Flex instrument. All RNA samples met the required quality parameters for sequencing, including a minimum concentration of 30 ng/μL, an A260/280 ratio above 2.00, and an RIN value above 7.00.

Next-generation sequencing (NGS) libraries were prepared with the Illumina^®®^ Stranded Total RNA Prep, Ligation with Ribo-Zero Plus (catalog number 20040529, San Diego, CA, USA) kit. Sequencing was conducted on a Novaseq 6000 instrument using paired-end 150-base-pair reads.

### 4.10. Transcriptome Analysis

#### 4.10.1. Gene Expression Quantification

RNA-Seq data obtained from *PLEC 1d* KD and NC MDA-T41 cells were subjected to quality control analysis with FastQC [50]. FastQ reads were mapped to the latest human Ensembl reference transcriptome (GRCh38) using the pseudoalignment program Kallisto (v0.46.2) [51], which is used to quantify transcript abundance. The R package Tximport (v1.38.2) was used to import transcript-level quantifications into a counting matrix [51,52]. A transcript-to-gene mapping file (tx2gene) was then passed to tximport to aggregate transcript abundances at the gene level. Gene annotations were retrieved using the biomaRt (v2.66.2) package by mapping Ensembl gene or transcript IDs to gene symbols [53].

#### 4.10.2. Differential Gene Expression Analysis

Differential expression analysis was performed using the DESeq2 R package (v1.46.0) based on tximport-derived count data, comparing NC- and *PLEC 1d*-suppressed MDA-T41 cells at both the gene and transcript levels [54]. Raw *p*-values were adjusted for multiple testing using the Benjamini–Hochberg procedure to estimate the false discovery rate (FDR). However, no genes remained statistically significant after FDR correction, most likely due to the limited number of biological replicates and the modest transcriptional effect size associated with partial isoform-specific *PLEC 1d* KD. Therefore, the RNA-seq analysis was interpreted as an exploratory, hypothesis-generating analysis rather than a definitive genome-wide discovery analysis. Differentially expressed genes (DEGs) were identified using a *p*-value < 0.05 as the cut-off criterion. Genes with log_2_ (fold change) > 0 were considered upregulated, whereas genes with log_2_ (fold change) < 0 were considered downregulated. Importantly, selected candidate genes emerging from this exploratory transcriptomic analysis were further evaluated by independent RT-qPCR validation and, where technically feasible, by Western blotting.

### 4.11. Pathway Enrichment Analyses

Molecular pathways associated with significant DEGs were identified using the enrichment analysis tool DAVID (v2025_2), based on the Kyoto Encyclopedia of Genes and Genomes (KEGG) and Reactome databases [55]. *p*-values were calculated using Fisher’s exact test, and Benjamini–Hochberg correction was applied to control FDR.

## Data Availability

The original contributions presented in this study are included in the article/Supplementary Materials. Further inquiries can be directed to the corresponding author.

## Supplementary Materials

The following supporting information can be downloaded at https://www.mdpi.com/article/10.3390/ijms27146339/s1.
