# Supplementary material for: Plectin 1d Isoform as a Potential Regulator of Metastatic Progression in Papillary Thyroid Carcinoma
Source: Int J Mol Sci. 2026 Jul 16;27(14):6339. doi: 10.3390/ijms27146339 (PMC13410127; doi:10.3390/ijms27146339)
Supplement: Supplementary file 1 [file ijms-27-06339-s001.zip › ijms-4386695-supplementary.pdf]

Table S1. Primer sequences used in this study.

Gene/Isoform	Primer Sequences	Amplicon Size (bp)	Application
PLEC 1	**F:** 5’-GTGCCTGCTACCACCCAG-3’<br>**R:** 5’-GGTGCTTGTTGACCCACTTG-3’	115	RT-qPCR
PLEC 1a	**F:** 5’-GGACAACCTGTACCTGGCT-3’<br>**R:** 5’-GGTGCTTGTTGACCCACTTG-3’	101	RT-qPCR
PLEC 1b	**F:** 5’-CTGTTTCCCTCCCTGGTGG-3’<br>**R:** 5’-GGTGCTTGTTGACCCACTTG-3’	148	RT-qPCR
PLEC 1c	**F:** 5’-CCTGGAACCTTGGGAAAACG-3’<br>**R:** 5’-GGTGCTTGTTGACCCACTTG-3’	159	RT-qPCR
PLEC 1d	**F:** 5’-TGACCTCCCACACCCCTG-3’<br>**R1:** 5’-GGTGCTTGTTGACCCACTTG-3’	129	RT-PCR/<br>RT-qPCR
PLEC 1e	**F:** 5’-AGCCATCCAGAACGAGATCA-3’<br>**R:** 5’-ATCGCGGAGGTCTTCATACA-3’	136	RT-qPCR
PLEC 1f	**F:** 5’-CCGACGAGCAGGACTTCAT-3’<br>**R:** 5’-ATCGCGGAGGTCTTCATACA-3’	158	RT-qPCR
PLEC 1g	**F:** 5’-GAGGGAGGTCTTGCTGGAG-3’<br>**R:** 5’-GGTGCTTGTTGACCCACTTG-3’	146	RT-qPCR
GAPDH	**F:** 5' -CAGGGCTGCTTTTAACTCTGGTA- 3'<br>**R:** 5' -TTGATGACAAGCTTCCCGTTCT- 3'	150	RT-qPCR
ACTB	**F:** 5’-AATCTGGCACCACACCTTCT-3’<br>**R:** 5’-AGCCTGGATAGCAACGTACA-3’	163	RT-qPCR
LAMA4	**F:** 5’-GGCCCAGAAGATGCTTGAAG- 3’<br>**R:** 5’-GACGACAGGAAACAGAGTGC- 3’	157	RT-qPCR
MMP1	**F:** 5’ -CGACAGAGATGAAGTCCGGT- 3’<br>**R:** 5’-TCTAGGGAAGCCAAAGGAGC- 3’	112	RT-qPCR
HEY1	**F:** 5’-TCGGCTCTAGGTTCCATGTC- 3’<br>**R:** 5’-GCCTTCTCAGCTCAGACAAA- 3’	124	RT-qPCR
